# Poly[(μ-β-hexa­cosa­oxidoocta­molybdato)tetra­kis­[3-(2-pyrid­yl)pyrazole]­dizinc(II)]

**DOI:** 10.1107/S1600536810025286

**Published:** 2010-07-03

**Authors:** Lujiang Hao, Bo Liu

**Affiliations:** a Shandong Provincial Key Laboratory of Microbial Engineering, Shandong Institute of Light Industry, Jinan 250353, People’s Republic of China

## Abstract

In the hydro­thermally prepared title compound, [Mo_8_Zn_2_O_26_(C_8_H_7_N_3_)_4_]_*n*_ or {[Zn(C_8_H_7_N_3_)_2_]_2_(Mo_8_O_26_)}_*n*_, the Zn^II^ atom is coordinated by two *N*,*N*′-bidentate 3-(2-pyrid­yl)pyrazole ligands and two O atoms from adjacent octa­molybdate polyanions, generating a distorted *cis*-ZnO_2_N_4_ octa­hedral geometry for the divalent metal ion. The complete octa­molbydate unit is generated by crystallographic inversion symmetry. The polyhedral connectivity leads to [100] chains in the crystal and N—H⋯O and N—H⋯(O,O) hydrogen bonds help to consolidate the packing.

## Related literature

For background to polyoxidomolybdates, see: Pope & Müller (1991 ▶). For related structures, see: Artero & Proust (2000 ▶); Lee *et al.* (2002 ▶).
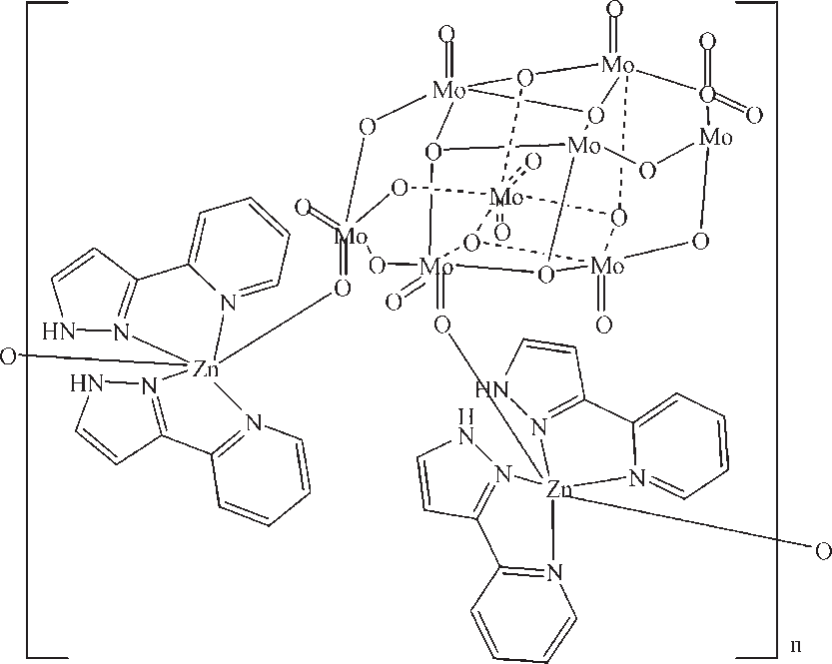


## Experimental

### 

#### Crystal data

[Mo_8_Zn_2_O_26_(C_8_H_7_N_3_)_4_]
*M* *_r_* = 947.46
Triclinic, 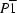

*a* = 10.0791 (8) Å
*b* = 11.5339 (10) Å
*c* = 11.6078 (10) Å
α = 89.007 (1)°
β = 74.731 (1)°
γ = 74.623 (1)°
*V* = 1253.19 (18) Å^3^
*Z* = 2
Mo *K*α radiation
μ = 2.97 mm^−1^
*T* = 296 K
0.12 × 0.10 × 0.08 mm

#### Data collection

Bruker APEXII CCD diffractometer
Absorption correction: multi-scan (*SADABS*; Bruker, 2001 ▶) *T*_min_ = 0.717, *T*_max_ = 0.797
8795 measured reflections
4353 independent reflections
3927 reflections with *I* > 2σ(*I*)
*R*_int_ = 0.015

#### Refinement

*R*[*F* ^2^ > 2σ(*F* ^2^)] = 0.019
*wR*(*F* ^2^) = 0.058
*S* = 1.00
4353 reflections
361 parameters
H-atom parameters constrained
Δρ_max_ = 0.50 e Å^−3^
Δρ_min_ = −0.46 e Å^−3^

### 

Data collection: *APEX2* (Bruker, 2004 ▶); cell refinement: *SAINT-Plus* (Bruker, 2001 ▶); data reduction: *SAINT-Plus*; program(s) used to solve structure: *SHELXS97* (Sheldrick, 2008 ▶); program(s) used to refine structure: *SHELXL97* (Sheldrick, 2008 ▶); molecular graphics: *SHELXTL* (Sheldrick, 2008 ▶); software used to prepare material for publication: *SHELXTL*.

## Supplementary Material

Crystal structure: contains datablocks global, I. DOI: 10.1107/S1600536810025286/hb5511sup1.cif

Structure factors: contains datablocks I. DOI: 10.1107/S1600536810025286/hb5511Isup2.hkl

Additional supplementary materials:  crystallographic information; 3D view; checkCIF report

## Figures and Tables

**Table 1:** Selected bond lengths (Å)

Zn1—N1	2.081 (3)
Zn1—N3	2.196 (2)
Zn1—N5	2.065 (2)
Zn1—N6	2.181 (2)
Zn1—O5^i^	2.104 (2)
Zn1—O13	2.252 (2)

Symmetry code: (i) 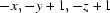.

**Table 2:** Hydrogen-bond geometry (Å, °)

*D*—H⋯*A*	*D*—H	H⋯*A*	*D*⋯*A*	*D*—H⋯*A*
N2—H2*A*⋯O1^i^	0.86	2.13	2.835 (3)	139
N4—H4⋯O6	0.86	2.38	3.094 (4)	141
N4—H4⋯O10^ii^	0.86	2.53	3.097 (3)	124

Symmetry codes: (i) 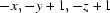; (ii) 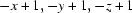.

**Fig. 1.**
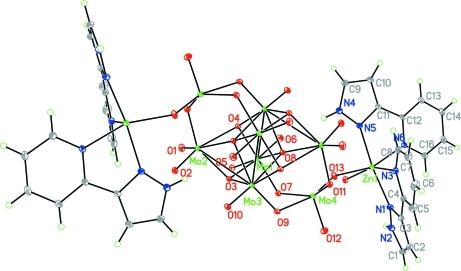
The building blocks of (I) with displacement ellipsoids drawn at the 30% probability level; H atoms are given as spheres of arbitrary radius.

**Fig. 2.**
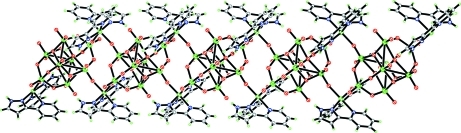
The crystal packing of (I) displayed with N—H···O hydrogen bonds as dashed lines.

**Fig. 3.**
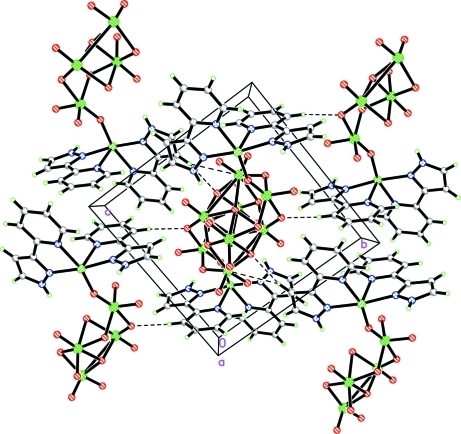
The chain structure.

## supplementary crystallographic information

### Comment

The design and synthesis of polyoxometalates has attracted continuous research interest not only because of their appealing structural and topological novelties, but also due to their interesting optical, electronic, magnetic, and catalytic properties, as well as their potential medical applications (Pope & Müller, 1991). Here, we describe the synthesis and structural characterization of the title compound.

As shown in Figure 1, As shown in Figure 1 and 2, the hexa-coordinated zinc cations act as a bridge to link two neighboring octamolybdate polyanions *via* terminal oxygen atoms, which are further chelated by two 3-(2-pyridyl)pyrazole ligands *via* four nitrogen atoms. The Zn—O and Zn—N distances are in the range of 2.104 (2)—2.252 (2) and 2.065 (2)—2.196 (2) Å, respectively.

The octamolybdate polyanion (Mo_8_O_26_)^2-^ shows a B configuration with a center of symmetry, which can be bisected into two [(/m5-O)(Mo4O12)]^2-^ planar subunits by Mo—O breaking bonds with the related lengths in the range of 2.26–2.39 Å, similar to previously reported isolated clusters (Lee *et al.*, 2002). The [(/m5-O)(Mo4O12)]^2-^ plane could be considered as one Mo atom protrudes outward from the other four Mo constituted planar. There are two types of Mo—-O bonds in octamolybdate polyanion: terminal Mo—O, and bridging /m2-O—Mo, /m3-O—Mo, and /m5-O—Mo bonds. The related bond distances vary from the shortest, 1.690 (2) Å for one of the terminal Mo—O bonds, to the longest 2.389 (2) Å for one of the bonds to the unusual /m5-O atom that sits in the 4Mo plane near the center of each Mo—O moiety.

In addition, it is noteworthy that the multipoint hydrogen-bonding links also exist between the hydrogen atoms from organic amines and the cluster of the surface oxygen atoms from the wave-like chains; this may make a contribution to stabilizing the chain structures, shown in figure 3.

### Experimental

The synthesis was performed in a 25-ml Teflon-lined stainless steel vessel. MoO_3_ (1 mmol, 0.144 g), zinc(II) acetate dihydrate (0.2 mmol, 0.044 g), 3-(2-pyridyl)pyrazole (0.35 mmol, 0.05 g), and H_2_O (14 ml) were mixed and heated to 423 K for three days.   Upon cooling, colourless blocks of (I) were recovered by vacuum filtration.

### Refinement

All hydrogen atoms bound to aromatic carbon atoms were refined in calculated positions using a riding model with a C—H distance of 0.93 Å and *U*_iso_ = 1.2*U*_eq_(C). The hydrogen atoms bound to N atoms were refined in calculated positions using a riding model with a N—H distance of 0.86 Å and *U*_iso_ = 1.2*U*_eq_(C).

### Crystal data

**Table:**

[Mo_8_Zn_2_O_26_(C_8_H_7_N_3_)_4_]	*Z* = 2
*M**_r_* = 947.46	*F*(000) = 908
Triclinic, *P*1	*D*_x_ = 2.511 Mg m^−^^3^
Hall symbol: -P 1	Mo *K*α radiation, λ = 0.71073 Å
*a* = 10.0791 (8) Å	Cell parameters from 6222 reflections
*b* = 11.5339 (10) Å	θ = 2.2–27.4°
*c* = 11.6078 (10) Å	µ = 2.97 mm^−^^1^
α = 89.007 (1)°	*T* = 296 K
β = 74.731 (1)°	Block, colorless
γ = 74.623 (1)°	0.12 × 0.10 × 0.08 mm
*V* = 1253.19 (18) Å^3^	

### Data collection

**Table:**

Bruker APEXII CCD diffractometer	4353 independent reflections
Radiation source: fine-focus sealed tube	3927 reflections with *I* > 2σ(*I*)
graphite	*R*_int_ = 0.015
phi and ω scans	θ_max_ = 25.0°, θ_min_ = 2.2°
Absorption correction: multi-scan (*SADABS*; Bruker, 2001)	*h* = −11→11
*T*_min_ = 0.717, *T*_max_ = 0.797	*k* = −13→13
8795 measured reflections	*l* = −13→13

### Refinement

**Table:**

Refinement on *F*^2^	Primary atom site location: structure-invariant direct methods
Least-squares matrix: full	Secondary atom site location: difference Fourier map
*R*[*F*^2^ > 2σ(*F*^2^)] = 0.019	Hydrogen site location: inferred from neighbouring sites
*wR*(*F*^2^) = 0.058	H-atom parameters constrained
*S* = 1.00	*w* = 1/[σ^2^(*F*_o_^2^) + (0.038*P*)^2^ + 0.1*P*] where *P* = (*F*_o_^2^ + 2*F*_c_^2^)/3
4353 reflections	(Δ/σ)_max_ = 0.001
361 parameters	Δρ_max_ = 0.50 e Å^−^^3^
0 restraints	Δρ_min_ = −0.46 e Å^−^^3^

### Special details

**Table:**

Geometry. All e.s.d.'s (except the e.s.d. in the dihedral angle between two l.s. planes) are estimated using the full covariance matrix. The cell e.s.d.'s are taken into account individually in the estimation of e.s.d.'s in distances, angles and torsion angles; correlations between e.s.d.'s in cell parameters are only used when they are defined by crystal symmetry. An approximate (isotropic) treatment of cell e.s.d.'s is used for estimating e.s.d.'s involving l.s. planes.
Refinement. Refinement of *F*^2^ against ALL reflections. The weighted *R*-factor *wR* and goodness of fit *S* are based on *F*^2^, conventional *R*-factors *R* are based on *F*, with *F* set to zero for negative *F*^2^. The threshold expression of *F*^2^ > σ(*F*^2^) is used only for calculating *R*-factors(gt) *etc*. and is not relevant to the choice of reflections for refinement. *R*-factors based on *F*^2^ are statistically about twice as large as those based on *F*, and *R*- factors based on ALL data will be even larger.

### Fractional atomic coordinates and isotropic or equivalent isotropic displacement parameters (Å^2^)

**Table:**

	*x*	*y*	*z*	*U*_iso_*/*U*_eq_	
C1	0.0264 (4)	−0.0590 (3)	0.3713 (3)	0.0412 (8)	
H1	−0.0334	−0.1045	0.4112	0.049*	
C2	0.1715 (4)	−0.0950 (3)	0.3361 (3)	0.0423 (8)	
H2	0.2305	−0.1689	0.3471	0.051*	
C3	0.2132 (3)	0.0033 (3)	0.2795 (3)	0.0308 (7)	
C4	0.3522 (3)	0.0215 (3)	0.2193 (3)	0.0325 (7)	
C5	0.4798 (4)	−0.0599 (3)	0.2184 (4)	0.0510 (10)	
H5	0.4822	−0.1305	0.2587	0.061*	
C6	0.6048 (4)	−0.0338 (4)	0.1556 (4)	0.0607 (11)	
H6	0.6928	−0.0862	0.1550	0.073*	
C7	0.5986 (4)	0.0683 (3)	0.0952 (4)	0.0529 (10)	
H7	0.6820	0.0845	0.0499	0.063*	
C8	0.4674 (4)	0.1478 (3)	0.1015 (3)	0.0404 (8)	
H8	0.4636	0.2186	0.0613	0.048*	
C9	0.2980 (4)	0.5417 (3)	0.0625 (3)	0.0411 (8)	
H9	0.3472	0.6002	0.0569	0.049*	
C10	0.2574 (4)	0.4981 (3)	−0.0278 (3)	0.0411 (8)	
H10	0.2728	0.5205	−0.1065	0.049*	
C11	0.1880 (3)	0.4130 (3)	0.0236 (3)	0.0289 (7)	
C12	0.1196 (3)	0.3344 (3)	−0.0244 (3)	0.0281 (7)	
C13	0.1012 (4)	0.3425 (3)	−0.1379 (3)	0.0379 (8)	
H13	0.1345	0.3977	−0.1891	0.045*	
C14	0.0328 (4)	0.2675 (3)	−0.1741 (3)	0.0437 (9)	
H14	0.0176	0.2720	−0.2499	0.052*	
C15	−0.0133 (4)	0.1852 (3)	−0.0965 (3)	0.0445 (9)	
H15	−0.0591	0.1331	−0.1197	0.053*	
C16	0.0094 (3)	0.1815 (3)	0.0150 (3)	0.0382 (8)	
H16	−0.0224	0.1264	0.0671	0.046*	
Mo1	0.19711 (2)	0.56897 (2)	0.54612 (2)	0.02158 (8)	
Mo2	0.39657 (2)	0.68604 (2)	0.68548 (2)	0.02263 (8)	
Mo3	0.50488 (2)	0.40073 (2)	0.61961 (2)	0.01958 (8)	
Mo4	0.30016 (3)	0.27733 (2)	0.48179 (2)	0.02400 (8)	
N1	0.0993 (3)	0.0933 (2)	0.2815 (2)	0.0324 (6)	
N2	−0.0148 (3)	0.0536 (2)	0.3381 (2)	0.0381 (7)	
H2A	−0.1023	0.0953	0.3510	0.046*	
N3	0.3455 (3)	0.1258 (2)	0.1639 (2)	0.0305 (6)	
N4	0.2540 (3)	0.4845 (2)	0.1601 (2)	0.0366 (6)	
H4	0.2672	0.4973	0.2285	0.044*	
N5	0.1867 (3)	0.4047 (2)	0.1388 (2)	0.0305 (6)	
N6	0.0758 (3)	0.2545 (2)	0.0517 (2)	0.0297 (6)	
O1	0.2356 (2)	0.7884 (2)	0.7426 (2)	0.0368 (5)	
O2	0.4653 (2)	0.64914 (19)	0.80341 (19)	0.0331 (5)	
O3	0.33613 (19)	0.53414 (17)	0.67987 (16)	0.0213 (4)	
O4	0.32825 (19)	0.67595 (16)	0.51042 (17)	0.0225 (4)	
O5	0.0610 (2)	0.65481 (19)	0.65921 (19)	0.0317 (5)	
O6	0.1412 (2)	0.5936 (2)	0.42044 (19)	0.0339 (5)	
O7	0.17612 (19)	0.41424 (17)	0.58213 (17)	0.0254 (4)	
O8	0.41746 (19)	0.44996 (16)	0.44765 (16)	0.0211 (4)	
O9	0.4158 (2)	0.28943 (18)	0.62220 (17)	0.0266 (5)	
O10	0.5745 (2)	0.37582 (19)	0.73826 (18)	0.0297 (5)	
O11	0.4944 (2)	0.21251 (17)	0.39605 (18)	0.0273 (5)	
O12	0.2475 (2)	0.1589 (2)	0.5449 (2)	0.0420 (6)	
O13	0.2277 (2)	0.3041 (2)	0.36228 (19)	0.0341 (5)	
Zn1	0.13422 (4)	0.25600 (3)	0.21960 (3)	0.02740 (10)	

### Atomic displacement parameters (Å^2^)

**Table:**

	*U*^11^	*U*^22^	*U*^33^	*U*^12^	*U*^13^	*U*^23^
C1	0.048 (2)	0.044 (2)	0.040 (2)	−0.0261 (17)	−0.0111 (17)	0.0084 (16)
C2	0.046 (2)	0.0324 (18)	0.048 (2)	−0.0101 (16)	−0.0131 (17)	0.0084 (16)
C3	0.0344 (17)	0.0280 (16)	0.0300 (17)	−0.0041 (13)	−0.0131 (14)	0.0023 (13)
C4	0.0336 (17)	0.0262 (16)	0.0331 (18)	−0.0012 (13)	−0.0078 (14)	−0.0032 (13)
C5	0.036 (2)	0.040 (2)	0.067 (3)	0.0012 (16)	−0.0104 (19)	0.0034 (18)
C6	0.032 (2)	0.059 (3)	0.083 (3)	−0.0005 (18)	−0.012 (2)	−0.009 (2)
C7	0.032 (2)	0.060 (3)	0.060 (3)	−0.0160 (19)	0.0035 (18)	−0.014 (2)
C8	0.043 (2)	0.0435 (19)	0.0363 (19)	−0.0196 (16)	−0.0052 (16)	−0.0066 (16)
C9	0.0412 (19)	0.046 (2)	0.042 (2)	−0.0223 (16)	−0.0122 (16)	0.0092 (17)
C10	0.046 (2)	0.047 (2)	0.0305 (18)	−0.0184 (17)	−0.0050 (16)	0.0082 (16)
C11	0.0260 (15)	0.0356 (17)	0.0223 (16)	−0.0061 (13)	−0.0043 (13)	0.0060 (13)
C12	0.0264 (15)	0.0320 (16)	0.0208 (15)	−0.0024 (13)	−0.0029 (12)	0.0010 (13)
C13	0.0412 (19)	0.047 (2)	0.0242 (17)	−0.0114 (16)	−0.0073 (15)	0.0019 (15)
C14	0.044 (2)	0.060 (2)	0.0270 (18)	−0.0108 (18)	−0.0121 (16)	−0.0042 (17)
C15	0.043 (2)	0.057 (2)	0.036 (2)	−0.0182 (17)	−0.0093 (16)	−0.0108 (17)
C16	0.0377 (19)	0.0397 (18)	0.0362 (19)	−0.0122 (15)	−0.0060 (15)	0.0000 (15)
Mo1	0.01351 (13)	0.02965 (14)	0.02125 (14)	−0.00523 (10)	−0.00454 (10)	−0.00102 (11)
Mo2	0.01700 (13)	0.02952 (14)	0.02131 (14)	−0.00717 (10)	−0.00387 (10)	−0.00267 (11)
Mo3	0.01706 (13)	0.02553 (14)	0.01692 (14)	−0.00626 (10)	−0.00557 (10)	0.00432 (10)
Mo4	0.02013 (14)	0.03132 (15)	0.02320 (15)	−0.01142 (11)	−0.00577 (11)	0.00101 (11)
N1	0.0256 (14)	0.0352 (14)	0.0321 (15)	−0.0045 (11)	−0.0044 (11)	0.0072 (12)
N2	0.0281 (14)	0.0484 (17)	0.0360 (16)	−0.0103 (13)	−0.0057 (12)	0.0060 (13)
N3	0.0287 (14)	0.0313 (14)	0.0297 (14)	−0.0063 (11)	−0.0065 (12)	−0.0038 (11)
N4	0.0402 (16)	0.0406 (16)	0.0350 (16)	−0.0123 (13)	−0.0190 (13)	0.0053 (13)
N5	0.0354 (15)	0.0327 (14)	0.0291 (14)	−0.0136 (12)	−0.0142 (12)	0.0077 (11)
N6	0.0308 (14)	0.0314 (13)	0.0254 (14)	−0.0067 (11)	−0.0068 (11)	−0.0008 (11)
O1	0.0228 (11)	0.0454 (13)	0.0374 (13)	−0.0042 (10)	−0.0043 (10)	−0.0099 (11)
O2	0.0352 (12)	0.0417 (13)	0.0285 (12)	−0.0151 (10)	−0.0138 (10)	−0.0011 (10)
O3	0.0159 (9)	0.0299 (10)	0.0185 (10)	−0.0075 (8)	−0.0037 (8)	−0.0001 (8)
O4	0.0180 (10)	0.0262 (10)	0.0225 (10)	−0.0049 (8)	−0.0055 (8)	0.0011 (8)
O5	0.0178 (10)	0.0401 (12)	0.0350 (13)	−0.0079 (9)	−0.0027 (9)	−0.0080 (10)
O6	0.0283 (12)	0.0461 (13)	0.0326 (12)	−0.0124 (10)	−0.0151 (10)	0.0049 (10)
O7	0.0188 (10)	0.0348 (11)	0.0227 (11)	−0.0119 (9)	−0.0010 (8)	−0.0010 (9)
O8	0.0180 (10)	0.0270 (10)	0.0184 (10)	−0.0067 (8)	−0.0045 (8)	0.0020 (8)
O9	0.0264 (11)	0.0325 (11)	0.0230 (11)	−0.0107 (9)	−0.0076 (9)	0.0069 (9)
O10	0.0295 (11)	0.0393 (12)	0.0229 (11)	−0.0090 (9)	−0.0120 (9)	0.0058 (9)
O11	0.0235 (11)	0.0266 (10)	0.0309 (12)	−0.0083 (8)	−0.0042 (9)	0.0008 (9)
O12	0.0392 (13)	0.0435 (14)	0.0482 (15)	−0.0243 (11)	−0.0070 (11)	0.0069 (11)
O13	0.0256 (11)	0.0489 (13)	0.0283 (12)	−0.0060 (10)	−0.0116 (10)	−0.0073 (10)
Zn1	0.02716 (19)	0.02776 (19)	0.02265 (19)	−0.00265 (14)	−0.00377 (15)	0.00391 (14)

### Geometric parameters (Å, °)

**Table:**

C1—N2	1.337 (4)	Mo1—O7	1.8795 (19)
C1—C2	1.359 (5)	Mo1—O4	2.0000 (19)
C1—H1	0.9300	Mo1—O8	2.2855 (18)
C2—C3	1.406 (4)	Mo1—O3	2.3153 (18)
C2—H2	0.9300	Mo2—O2	1.690 (2)
C3—N1	1.324 (4)	Mo2—O1	1.706 (2)
C3—C4	1.459 (4)	Mo2—O11^i^	1.8892 (19)
C4—N3	1.348 (4)	Mo2—O3	2.0089 (19)
C4—C5	1.375 (4)	Mo2—O8^i^	2.3154 (18)
C5—C6	1.384 (5)	Mo2—O4	2.3248 (19)
C5—H5	0.9300	Mo3—O10	1.6910 (19)
C6—C7	1.356 (6)	Mo3—O9	1.747 (2)
C6—H6	0.9300	Mo3—O3	1.9424 (18)
C7—C8	1.380 (5)	Mo3—O4^i^	1.9517 (19)
C7—H7	0.9300	Mo3—O8^i^	2.1294 (18)
C8—N3	1.337 (4)	Mo3—O8	2.3886 (18)
C8—H8	0.9300	Mo4—O12	1.682 (2)
C9—N4	1.334 (4)	Mo4—O13	1.718 (2)
C9—C10	1.369 (5)	Mo4—O11	1.8985 (19)
C9—H9	0.9300	Mo4—O7	1.9139 (19)
C10—C11	1.389 (4)	Mo4—O9	2.2644 (19)
C10—H10	0.9300	N1—N2	1.354 (3)
C11—N5	1.336 (4)	N2—H2A	0.8600
C11—C12	1.470 (4)	N4—N5	1.340 (3)
C12—N6	1.344 (4)	N4—H4	0.8600
C12—C13	1.377 (4)	O4—Mo3^i^	1.9517 (19)
C13—C14	1.371 (5)	O5—Zn1^ii^	2.104 (2)
C13—H13	0.9300	O8—Mo3^i^	2.1294 (18)
C14—C15	1.382 (5)	O8—Mo2^i^	2.3154 (18)
C14—H14	0.9300	O11—Mo2^i^	1.8892 (19)
C15—C16	1.370 (5)	Zn1—N1	2.081 (3)
C15—H15	0.9300	Zn1—N3	2.196 (2)
C16—N6	1.342 (4)	Zn1—N5	2.065 (2)
C16—H16	0.9300	Zn1—N6	2.181 (2)
Mo1—O6	1.691 (2)	Zn1—O5^ii^	2.104 (2)
Mo1—O5	1.721 (2)	Zn1—O13	2.252 (2)
			
N2—C1—C2	107.6 (3)	O8^i^—Mo2—O4	72.75 (6)
N2—C1—H1	126.2	O10—Mo3—O9	104.53 (10)
C2—C1—H1	126.2	O10—Mo3—O3	102.49 (9)
C1—C2—C3	105.3 (3)	O9—Mo3—O3	96.90 (9)
C1—C2—H2	127.3	O10—Mo3—O4^i^	100.83 (9)
C3—C2—H2	127.3	O9—Mo3—O4^i^	96.38 (9)
N1—C3—C2	110.1 (3)	O3—Mo3—O4^i^	149.23 (8)
N1—C3—C4	117.0 (3)	O10—Mo3—O8^i^	99.20 (9)
C2—C3—C4	132.9 (3)	O9—Mo3—O8^i^	156.24 (8)
N3—C4—C5	122.3 (3)	O3—Mo3—O8^i^	78.72 (7)
N3—C4—C3	114.3 (3)	O4^i^—Mo3—O8^i^	77.97 (8)
C5—C4—C3	123.4 (3)	O10—Mo3—O8	174.80 (9)
C4—C5—C6	118.1 (4)	O9—Mo3—O8	80.66 (7)
C4—C5—H5	121.0	O3—Mo3—O8	76.97 (7)
C6—C5—H5	121.0	O4^i^—Mo3—O8	77.98 (7)
C7—C6—C5	119.9 (4)	O8^i^—Mo3—O8	75.60 (8)
C7—C6—H6	120.0	O12—Mo4—O13	104.85 (11)
C5—C6—H6	120.0	O12—Mo4—O11	104.88 (10)
C6—C7—C8	119.3 (4)	O13—Mo4—O11	98.55 (9)
C6—C7—H7	120.3	O12—Mo4—O7	104.77 (10)
C8—C7—H7	120.3	O13—Mo4—O7	97.38 (9)
N3—C8—C7	121.7 (3)	O11—Mo4—O7	141.25 (8)
N3—C8—H8	119.2	O12—Mo4—O9	91.45 (9)
C7—C8—H8	119.2	O13—Mo4—O9	163.67 (9)
N4—C9—C10	107.2 (3)	O11—Mo4—O9	77.82 (8)
N4—C9—H9	126.4	O7—Mo4—O9	77.04 (8)
C10—C9—H9	126.4	C3—N1—N2	105.8 (2)
C9—C10—C11	105.1 (3)	C3—N1—Zn1	117.2 (2)
C9—C10—H10	127.5	N2—N1—Zn1	136.5 (2)
C11—C10—H10	127.5	C1—N2—N1	111.2 (3)
N5—C11—C10	110.5 (3)	C1—N2—H2A	124.4
N5—C11—C12	116.9 (3)	N1—N2—H2A	124.4
C10—C11—C12	132.5 (3)	C8—N3—C4	118.6 (3)
N6—C12—C13	122.7 (3)	C8—N3—Zn1	126.8 (2)
N6—C12—C11	114.6 (3)	C4—N3—Zn1	113.3 (2)
C13—C12—C11	122.6 (3)	N5—N4—C9	111.9 (3)
C14—C13—C12	118.6 (3)	N5—N4—H4	124.1
C14—C13—H13	120.7	C9—N4—H4	124.1
C12—C13—H13	120.7	C11—N5—N4	105.3 (2)
C13—C14—C15	119.2 (3)	C11—N5—Zn1	116.1 (2)
C13—C14—H14	120.4	N4—N5—Zn1	136.35 (19)
C15—C14—H14	120.4	C12—N6—C16	118.0 (3)
C14—C15—C16	119.1 (3)	C12—N6—Zn1	113.7 (2)
C14—C15—H15	120.4	C16—N6—Zn1	128.2 (2)
C16—C15—H15	120.4	Mo3—O3—Mo2	108.99 (8)
N6—C16—C15	122.3 (3)	Mo3—O3—Mo1	110.34 (8)
N6—C16—H16	118.8	Mo2—O3—Mo1	104.40 (8)
C15—C16—H16	118.8	Mo3^i^—O4—Mo1	108.87 (9)
O6—Mo1—O5	105.92 (10)	Mo3^i^—O4—Mo2	109.70 (8)
O6—Mo1—O7	102.36 (9)	Mo1—O4—Mo2	104.35 (8)
O5—Mo1—O7	100.55 (9)	Mo1—O5—Zn1^ii^	167.30 (12)
O6—Mo1—O4	96.01 (9)	Mo1—O7—Mo4	119.86 (10)
O5—Mo1—O4	100.20 (9)	Mo3^i^—O8—Mo1	93.40 (7)
O7—Mo1—O4	147.19 (8)	Mo3^i^—O8—Mo2^i^	92.63 (7)
O6—Mo1—O8	93.81 (9)	Mo1—O8—Mo2^i^	163.58 (9)
O5—Mo1—O8	159.90 (8)	Mo3^i^—O8—Mo3	104.40 (8)
O7—Mo1—O8	78.43 (7)	Mo1—O8—Mo3	97.00 (7)
O4—Mo1—O8	73.40 (7)	Mo2^i^—O8—Mo3	96.26 (6)
O6—Mo1—O3	163.48 (9)	Mo3—O9—Mo4	120.70 (9)
O5—Mo1—O3	87.50 (8)	Mo2^i^—O11—Mo4	120.57 (10)
O7—Mo1—O3	84.12 (7)	Mo4—O13—Zn1	155.18 (13)
O4—Mo1—O3	71.70 (7)	N5—Zn1—N1	172.65 (10)
O8—Mo1—O3	72.41 (7)	N5—Zn1—O5^ii^	98.46 (9)
O2—Mo2—O1	105.26 (11)	N1—Zn1—O5^ii^	88.41 (9)
O2—Mo2—O11^i^	102.35 (9)	N5—Zn1—N6	76.74 (10)
O1—Mo2—O11^i^	100.69 (10)	N1—Zn1—N6	99.31 (10)
O2—Mo2—O3	94.91 (9)	O5^ii^—Zn1—N6	102.52 (9)
O1—Mo2—O3	101.12 (10)	N5—Zn1—N3	98.82 (10)
O11^i^—Mo2—O3	147.40 (8)	N1—Zn1—N3	75.45 (10)
O2—Mo2—O8^i^	94.13 (9)	O5^ii^—Zn1—N3	155.52 (9)
O1—Mo2—O8^i^	160.27 (9)	N6—Zn1—N3	98.23 (9)
O11^i^—Mo2—O8^i^	78.24 (7)	N5—Zn1—O13	84.54 (9)
O3—Mo2—O8^i^	73.08 (7)	N1—Zn1—O13	98.92 (9)
O2—Mo2—O4	163.06 (9)	O5^ii^—Zn1—O13	83.37 (8)
O1—Mo2—O4	87.52 (9)	N6—Zn1—O13	160.98 (9)
O11^i^—Mo2—O4	85.74 (8)	N3—Zn1—O13	81.16 (8)
O3—Mo2—O4	71.34 (7)		

Symmetry codes: (i) −*x*+1, −*y*+1, −*z*+1; (ii) −*x*, −*y*+1, −*z*+1.

### Hydrogen-bond geometry (Å, °)

**Table:**

*D*—H···*A*	*D*—H	H···*A*	*D*···*A*	*D*—H···*A*
N2—H2A···O1^ii^	0.86	2.13	2.835 (3)	139
N4—H4···O6	0.86	2.38	3.094 (4)	141
N4—H4···O10^i^	0.86	2.53	3.097 (3)	124

Symmetry codes: (ii) −*x*, −*y*+1, −*z*+1; (i) −*x*+1, −*y*+1, −*z*+1.
